# Supplementary material for: Rapid and quantitative functional interrogation of human enhancer variant activity in live mice
Source: Nat Commun. 2025 Jan 6;16:409. doi: 10.1038/s41467-024-55500-7 (PMC11704014; doi:10.1038/s41467-024-55500-7)
Supplement: Supplementary file 1 — Supplementary Information [file 41467_2024_55500_MOESM1_ESM.pdf]

Supplementary Information

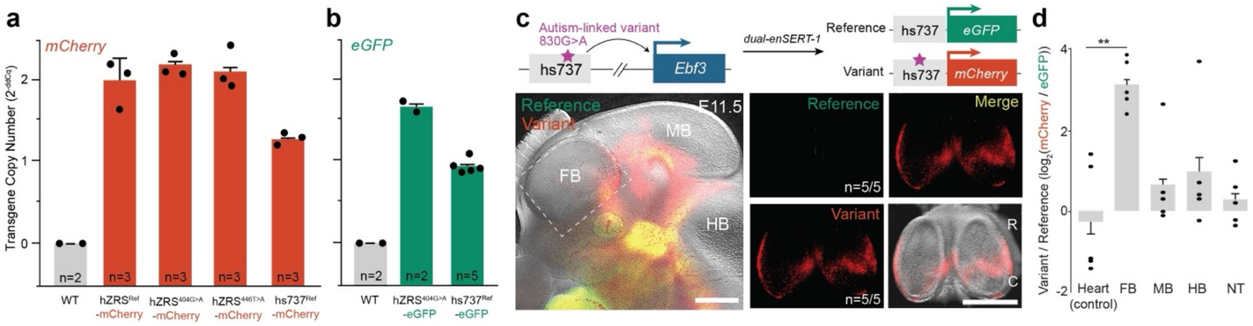

Supplementary Figure 1. **Transgene copy-number quantification for dual-enSERT-1 and simultaneous comparison of human reference and variant hs737 enhancer activities in the brain. (a, b)** Quantitative PCR plot for *mCherry* (a) and *eGFP* (b) transgene copy number across dual-enSERT-1 lines. Note equivalent copy number for each enhancer allele (i.e., two for hZRS and one for hs737). Data represented as mean ± SEM. Data points represent independent biological replicates (mice). (**c**) Representative images of transgenic $hs737^{ref}$-*eGFP*/$hs737^{830G>A}$-*mCherry* embryos at E11.5. A close-up of the dissected forebrain with separate and merged channels shown. R, rostral; C, caudal. (**d**) Plots quantifying fold-change (log2) difference in reporter intensity between variant and reference hs737 alleles. Two-sided paired t-tests: Forebrain (FB), *P* = 0.0043; Midbrain (MB), *P* = ns; Hindbrain (HB), *P* = ns; Neural Tube (NT), *P* = ns. Data represented as mean ± SEM. All scale bars, 500 µm. Data points represent independent biological replicates (n = 5 embryos). Source data are provided as a Source Data file.

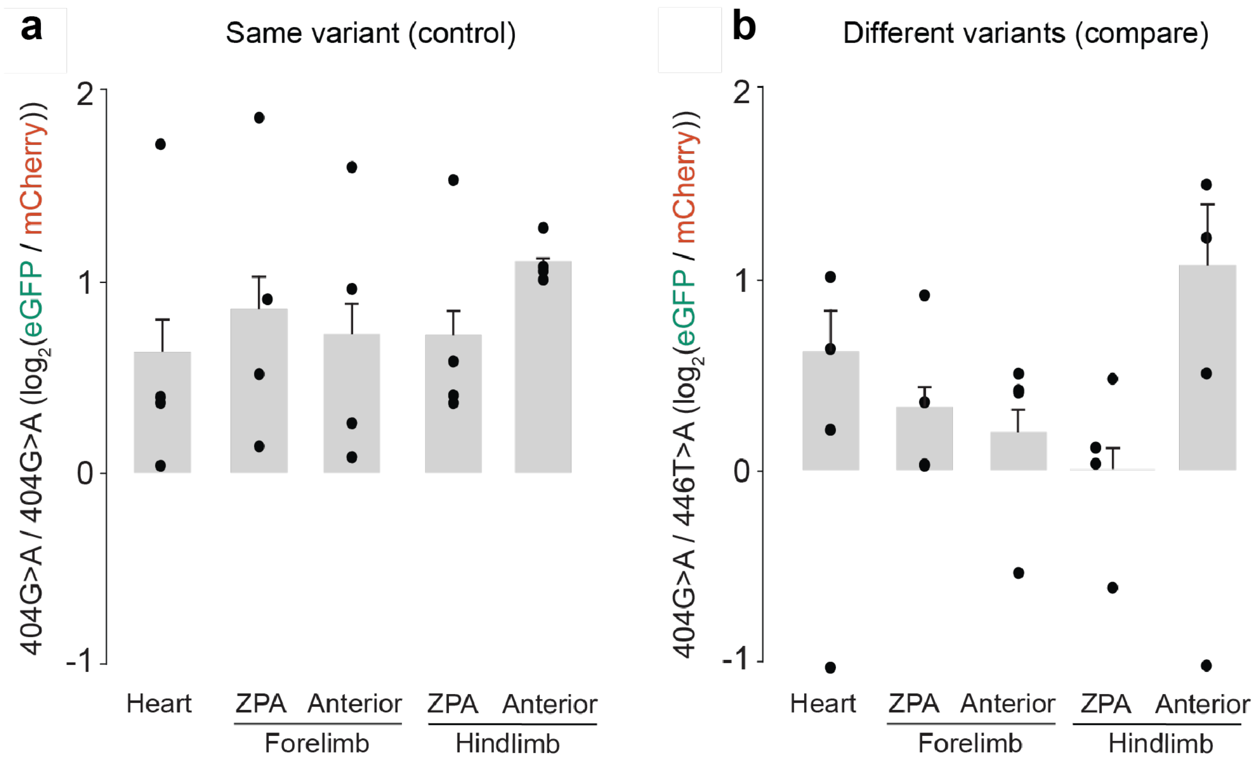

Supplementary Figure 2: Quantitative analysis of hZRS variant allele-driven reporter intensities. (**a**) Quantitative plot for fold-change ($\log_2$) difference in fluorescent reporter intensity between the same 404G>A variant allele from hZRS$^{404G>A}$-*mCherry*/hZRS$^{404G>A}$-*eGFP* embryos at E11.5. Two-sided paired t-tests vs. Heart: Forelimb ZPA, *P* = ns; Forelimb Anterior, *P* = ns; Hindlimb ZPA, *P* = ns; Hindlimb Anterior, *P* = ns. Data represented as mean ± SEM. Data points represent independent biological replicates (n=4 embryos). (**b**) Quantitative plot for fold-change ($\log_2$) difference in fluorescent reporter intensity between 404G>A and 446T>A variant alleles from hZRS$^{446T>A}$-*mCherry*/hZRS$^{404G>A}$-*eGFP* embryos at E11.5. Two-sided paired t-tests vs. Heart: Forelimb ZPA, *P* = ns; Forelimb Anterior, *P* = ns; Hindlimb ZPA, *P* = ns; Hindlimb Anterior, *P* = ns. Data represented as mean ± SEM. Data points represent independent biological replicates (n=4 embryos). Source data are provided as a Source Data file.

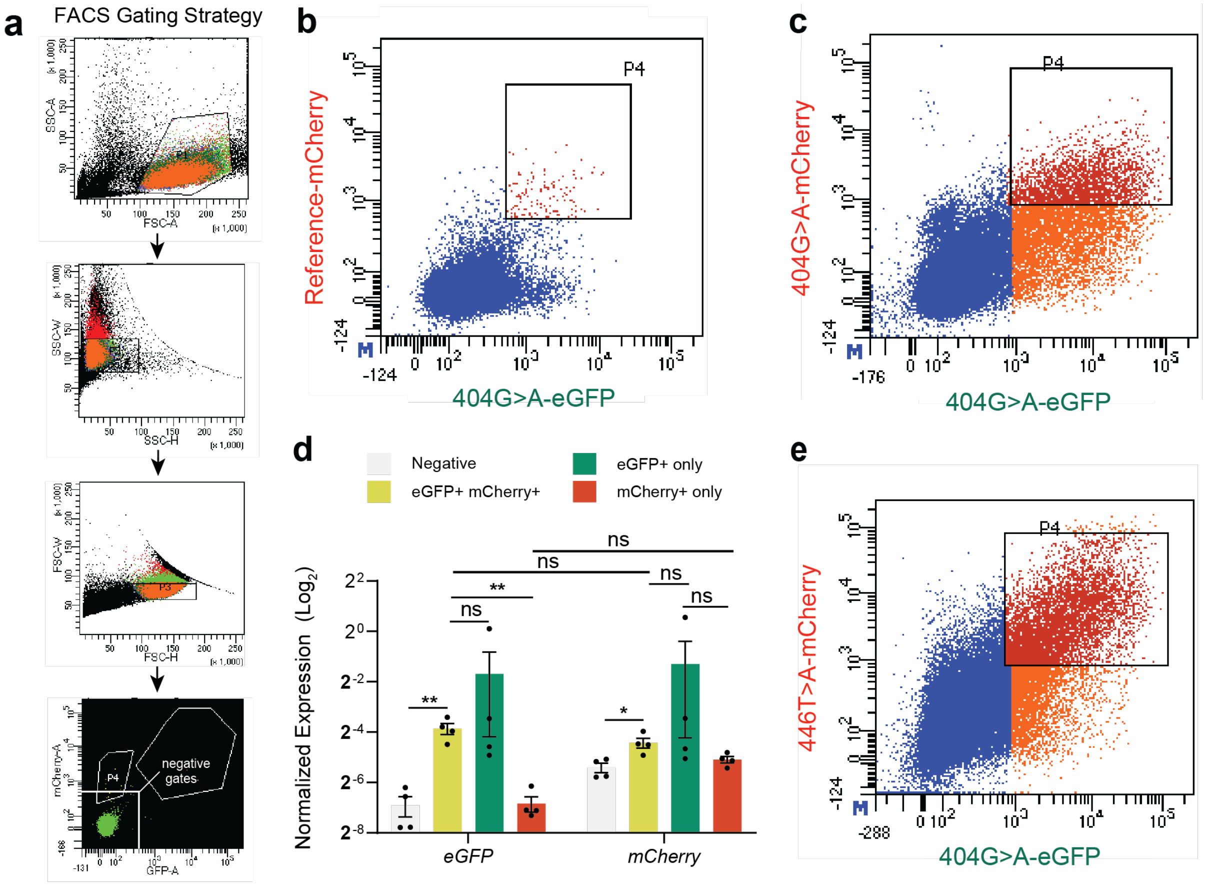

Supplementary Figure 3: Quantification of *eGFP* and *mCherry* in anterior hindlimbs of hZRS dual-enSERT-1 embryos by FACS and qPCR. (**a**) Sequential gating strategy for FACS-based analysis of dual fluorescence. Bottom panel shows forebrain cells from a dual-enSERT-1 embryo for gating mCherry and eGFP fluorescence. (**b**) Representative flow cytometry plots for anterior portion of E11.5 hindlimbs from hZRS^{ref}-*mCherry*/hZRS$^{404G>A}$-*eGFP.* (**c**) Example flow cytometry plots for anterior portion of E11.5 hindlimbs from hZRS$^{446T>A}$-*mCherry*/hZRS$^{404G>A}$-*eGFP.* (**d**) Absolute transcript levels of *eGFP* and *mCherry* normalized to *Gapdh* from anterior limb-sorted cells from hZRS$^{404G>A}$-*mCherry*/hZRS$^{404G>A}$-*eGFP* embryos. Student's t-test with two-tailed distribution and unequal variance (ns > 0.05, * < 0.05, ** < 0.01, *** < 0.001). Data points represent independent biological replicates (n=4 embryos). Data represented as mean ± SEM for all plots. (**e**) Sample flow cytometry plots for anterior portion of E11.5 hindlimbs from hZRS$^{404G>A}$-*mCherry*/hZRS$^{404G>A}$-*eGFP.*

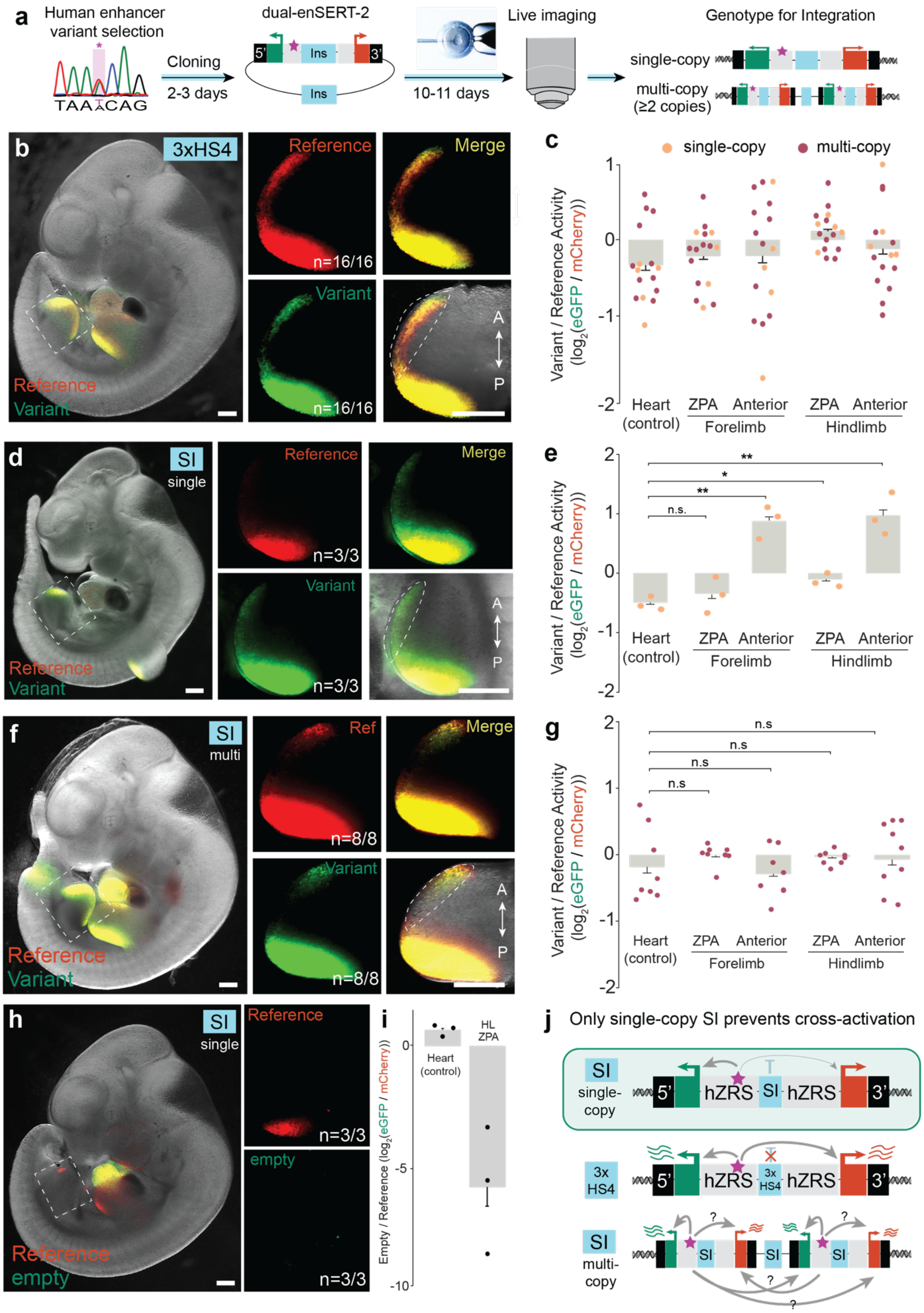
a
Human enhancer variant selection
TAAACAG
Cloning
2-3 days
dual-enSERT-2
5'
Ins
3'
Ins
10-11 days
Live imaging
Genotype for Integration
single-copy
multi-copy
(≥2 copies)
b
3xHS4
Reference
Merge
n=16/16
Variant
n=16/16
A
P
Reference
Variant
c
single-copy
multi-copy
Variant / Reference Activity
(log2(eGFP / mCherry))
1
0
-1
-2
Heart (control)
ZPA
Anterior
Forelimb
ZPA
Anterior
Hindlimb
d
SI
single
Reference
Merge
n=3/3
Variant
n=3/3
A
P
Reference
Variant
e
2
1
0
-1
-2
**
*
**
n.s.
Heart (control)
ZPA
Anterior
Forelimb
ZPA
Anterior
Hindlimb
f
SI
multi
Ref
Merge
n=8/8
Variant
n=8/8
A
P
Reference
Variant
g
n.s
n.s
n.s
n.s
Heart (control)
ZPA
Anterior
Forelimb
ZPA
Anterior
Hindlimb
h
SI
single
Reference
n=3/3
empty
n=3/3
Reference
empty
i
Empty / Reference (log2(eGFP / mCherry))
0
-5
-10
Heart (control)
HL ZPA
j
Only single-copy SI prevents cross-activation
SI
single-copy
5'
hZRS
SI
hZRS
3'
3x HS4
5'
hZRS
3x HS4
hZRS
3'
SI
multi-copy
SI
SI
SI
?
?
?
?

Supplementary Figure 4: Optimization of a synthetic insulator for dual-enSERT-2 that prevents transgene reporter cross-activation. (**a**) Schematic of the dual-enSERT-2 system with enhancer alleles driving *eGFP* or *mCherry* placed on the same transgene and separated by an insulator (Ins). Microscope objective cartoon reproduced courtesy of Augustin Carpaneto (http://sci-draw.io). (**b, c**) Sample image (b) and Fold-change (log_2) quantification of variant to reference allele fluorescent reporter intensity (c) in hZRS^{ref}-*mCherry*/3xHS4/hZRS$^{404G>A}$-*eGFP* embryos at E11.5. Panels on right show an expanded view of hindlimb. Scale bars, 500 μm. Data points represent independent biological replicates (n=5 single-copy embryos; n=11 multi-copy embryos). Two-sided paired t-tests vs. Heart: Forelimb ZPA, *P* = ns; Forelimb Anterior, *P* = ns; Hindlimb ZPA, *P* = ns; Hindlimb Anterior, *P* = ns. Data represented as mean ± SEM. (**d**) Sample images of single-copy embryos injected with hZRS^{ref}-*mCherry*/SI/ZRS$^{404G>A}$-*eGFP* construct with hindlimb highlighted by dashed box and higher-resolution images on right. SI, synthetic insulator. (**e**) Plot quantifying fold-change (log_2) difference in reporter intensity for single-copy integrants. Data points represent independent biological replicates (n=3 embryos). Two-sided paired t-test vs. Heart: Single-copy: Forelimb ZPA, *P* = ns; Forelimb Anterior, *P* = 0.00126; Hindlimb ZPA, *P* = 0.0110; Hindlimb Anterior, *P* = 0.00235. Data represented as mean ± SEM. (**f**) Sample images of multi-copy embryos injected with hZRS^{ref}-*mCherry*/SI/ZRS$^{404G>A}$-*eGFP* construct with hindlimb highlighted by dashed box and higher-resolution images on right. (**g**) Plot quantifying fold-change (log_2) difference in reporter intensity for multi-copy integrants. Data points represent independent biological replicates (n=8 embryos). Two-sided paired t-tests vs. Heart. Multi-copy: Forelimb ZPA, *P* = ns; Forelimb Anterior, *P* = ns; Hindlimb ZPA, *P* = ns; Hindlimb Anterior, *P* = ns. Data represented as mean ± SEM. (**h, i**) Representative fluorescent image (h) and fold-change (log_2) quantification of empty to reference allele fluorescent reporter intensity of E11.5 dual-enSERT-2 embryos injected with mZRS^{ref}-*mCherry*/SI/empty-*eGFP* construct. Data points represent independent biological replicates (n=3 embryos). Data represented as mean ± SEM. (**j**) Schematic summary depicting that only single-copy integrants of dual-enSERT-2 constructs containing the synthetic insulator are sufficient to prevent cross-activation of reporter transgenes. Source data are provided as a Source Data file.

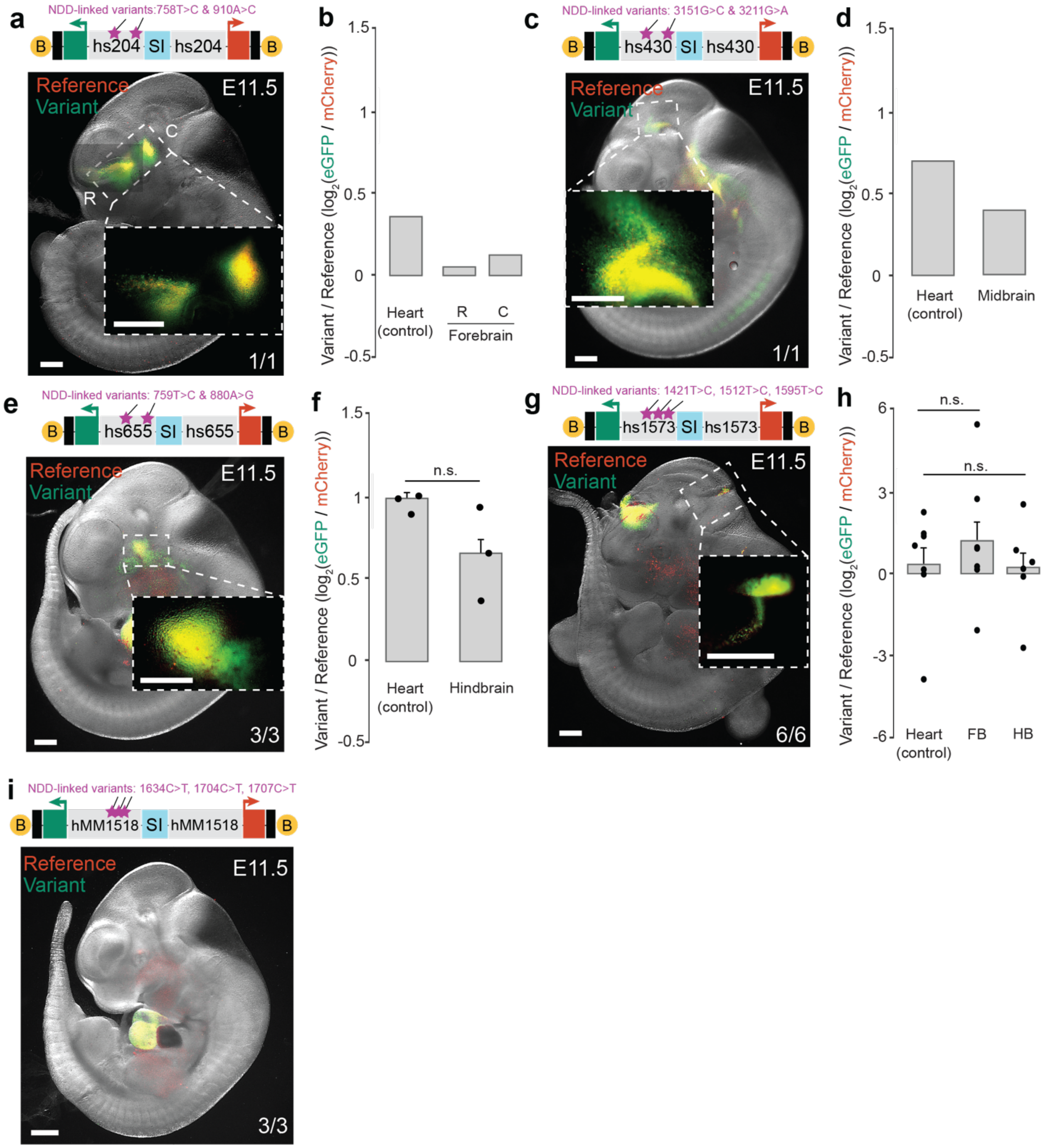

Supplementary Figure 5. Numerous patient variants linked to neurodevelopmental disorders show no effect on brain enhancer activity *in vivo*. (a-i) Fluorescent images for whole E11.5 embryos injected with B-hs204ref-*mCherry*/SI/hs204var-*eGFP*-B (a; n=1 embryo), B-hs430ref-*mCherry*/SI/hs430var-*eGFP*-B (c; n=1 embryo), B-hs655ref-*mCherry*/SI/hs655var-*eGFP*-B (e; n=3 embryos), B-hs1573ef-*mCherry*/SI/hs1573var-*eGFP*-B (g; n=6 embryos), and B-hMM1518ref-*mCherry*/SI/hMM1518var-*eGFP*-B (i; n=3 embryos). Plots quantifying ratios (log_2) of variant-driven *eGFP* to reference allele-driven *mCherry* for hs204 (b), hs430 (d), hs655 (f), hs1573 (h). Paired student t-test vs. Heart: All comparisons, not significant. Data represented as mean ± SEM. B, biotin; C, caudal; R, rostral. Embryos are independent biological replicates. Source data are provided as a Source Data file.

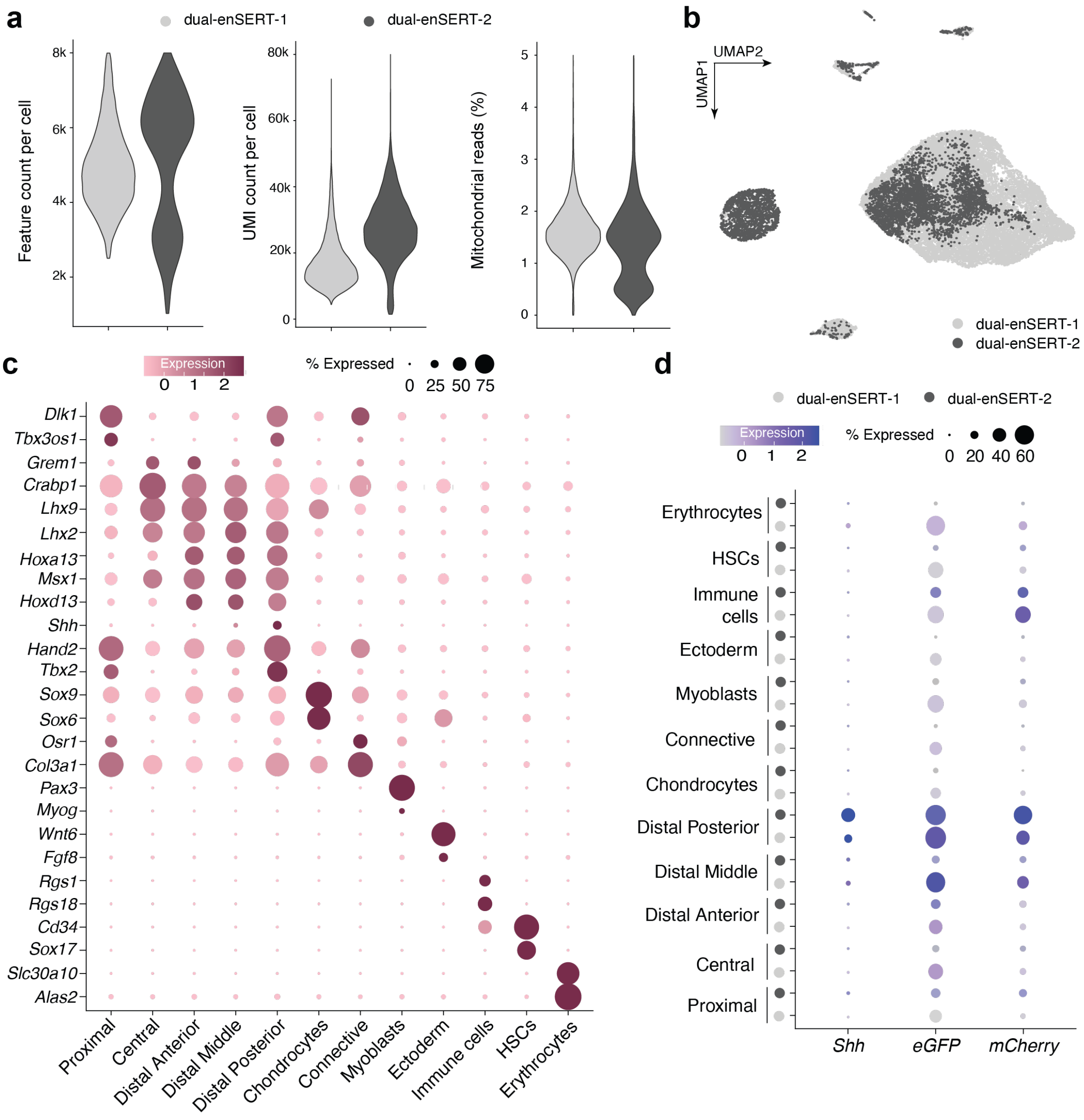

Supplementary Figure 6. Quality control for scRNA-seq from E11.5 hindlimb buds of dual-enSERT-1 and dual-enSERT-2 embryos. (a) Plots depicting feature and UMI counts and percent mitochondrial genes expressed, split by version of dual-enSERT. **(b)** UMAP plot of integrated dataset colored by dual-ensERT version. **(c)** Cell type marker expression across all clusters of the integrated dataset. (**d**) DotPlot depicts average expression and percent-expressing cells of the hZRS target gene *Shh*, *eGFP*, and *mCherry*, split by version of dual-enSERT. Dual-enSERT-1, light grey; dual-enSERT-2, dark grey.

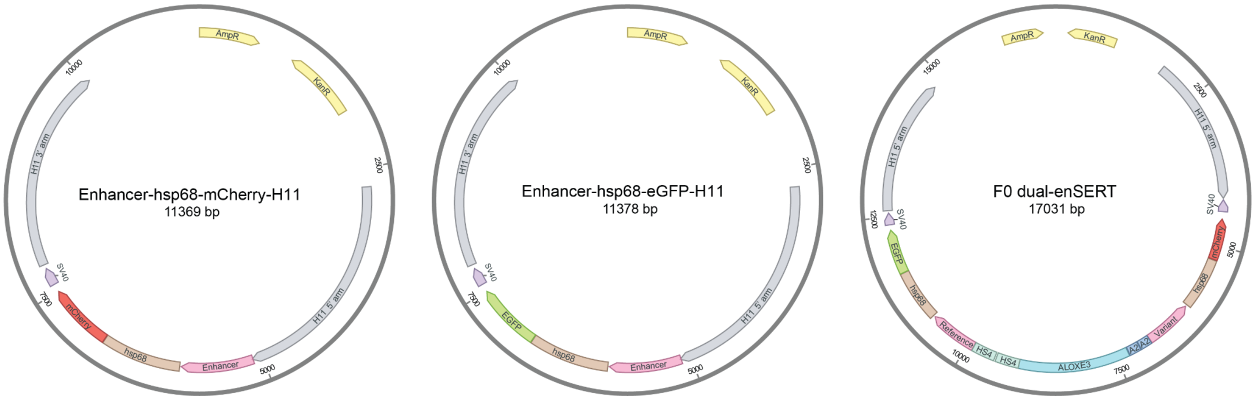

Supplementary Figure 7. Schematic representation of plasmids deposited by this study. Note that deposited versions of these plasmids are empty backbones without any enhancer sequences.

Supplementary Tables

Supplementary Table 1. Plasmids created and deposited by this study.

Plasmid name	Version	Addgene ID
PCR4-Hsp68::mCherry-H11 (empty vector)	*dual-enSERT-1*	#211940
PCR4-hZRS^{ref}-Hsp68::mCherry-H11	*dual-enSERT-1*	-
PCR4-hZRS$^{404G>A}$-Hsp68::mCherry-H11	*dual-enSERT-1*	-
PCR4-hZRS$^{446T>A}$-Hsp68::mCherry-H11	*dual-enSERT-1*	-
PCR4-hs737$^{830G>A}$-Hsp68::mCherry-H11	*dual-enSERT-1*	-
PCR4-Hsp68::eGFP-H11 (empty vector)	*dual-enSERT-1*	#211941
PCR4-hZRS$^{404G>A}$-Hsp68::eGFP-H11	*dual-enSERT-1*	-
PCR4-hs737^{ref}-Hsp68::eGFP-H11	*dual-enSERT-1*	-
PCR4-Hsp68::mCherry-3xHS4-Hsp68::eGFP-H11(empty vector)	*dual-enSERT-2*	-
PCR4-hZRS^{ref}-Hsp68::mCherry-3xHS4-hZRS$^{404G>A}$-Hsp68::eGFP-3xHS4-H11	*dual-enSERT-2*	-
PCR4-Hsp68::mCherry-SI-Hsp68::eGFP-H11-SI (empty backbone)	*dual-enSERT-2*	#211942
PCR4-hZRS^{ref}-Hsp68::mCherry-SI-hZRS$^{404G>A}$-Hsp68::eGFP-SI-H11	*dual-enSERT-2*	-
PCR4-Hsp68::mCherry-SI-Hsp68::eGFP-H11 (empty vector)	*dual-enSERT-2*	-
PCR4-hZRS^{ref}-Hsp68::mCherry-SI-hZRS$^{404G>A}$-Hsp68::eGFP-H11	*dual-enSERT-2*	-
PCR4-h737$^{830G>A}$-Hsp68::mCherry-SI-hs737^{ref}-Hsp68::eGFP-H11	*dual-enSERT-2*	-
PCR4-hs204^{ref}-Hsp68::mCherry-SI-hs204^{var}-Hsp68::eGFP-H11	*dual-enSERT-2*	-
PCR4-hs268^{ref}-Hsp68::mCherry-SI-hs268^{var}-Hsp68::eGFP-H11	*dual-enSERT-2*	-
PCR4-hs430^{ref}-Hsp68::mCherry-SI-hs430^{var}-Hsp68::eGFP-H11	*dual-enSERT-2*	-
PCR4-hs655^{ref}-Hsp68::mCherry-SI-hs655^{var}-Hsp68::eGFP-H11	*dual-enSERT-2*	-
PCR4-hs1573^{ref}-Hsp68::mCherry-SI-hs1573^{var}-Hsp68::eGFP-H11	*dual-enSERT-2*	-
PCR4-hs1791^{ref}-Hsp68::mCherry-SI-hs1791^{var}-Hsp68::eGFP-H11	*dual-enSERT-2*	-
PCR4-hMM1518^{ref}-Hsp68::mCherry-SI-hMM1518^{var}-Hsp68::eGFP-H11	*dual-enSERT-2*	-
