# Supplementary material for: Rapid and quantitative functional interrogation of human enhancer variant activity in live mice
Source: Nat Commun. 2025 Jan 6;16:409. doi: 10.1038/s41467-024-55500-7 (PMC11704014; doi:10.1038/s41467-024-55500-7)
Supplement: Supplementary file 3 — Description of Additional Supplementary Files [file 41467_2024_55500_MOESM3_ESM.pdf]

<u>Description of Additional Supplementary Files</u>

Supplementary Data 1: Genotyping and cross-activation results for *dual-enSERT-2* constructs. hMM, human ortholog of mouse enhancer.

Supplementary Data 2: Primers used in this study for cloning, nested PCR, and qPCR.

Supplementary Data 3: Barcode meta-data for single-cell transcriptomics of dual-enSERT E11.5 hindlimbs with human reference and variant ZRS alleles. dE1, dual-enSERT-1; dE2, dual-enSERT-2.
