# Supplementary material for: Rapid and quantitative functional interrogation of human enhancer variant activity in live mice
Source: Nat Commun. 2025 Jan 6;16:409. doi: 10.1038/s41467-024-55500-7 (PMC11704014; doi:10.1038/s41467-024-55500-7)
Supplement: Supplementary file 7 — Reporting Summary [file 41467_2024_55500_MOESM7_ESM.pdf]

nature portfolio

Corresponding author(s): Evgeny Kvon
Last updated by author(s): Nov 26, 2024

Reporting Summary

Nature Portfolio wishes to improve the reproducibility of the work that we publish. This form provides structure for consistency and transparency in reporting. For further information on Nature Portfolio policies, see our Editorial Policies and the Editorial Policy Checklist.

Statistics

For all statistical analyses, confirm that the following items are present in the figure legend, table legend, main text, or Methods section.

n/a	Confirmed	
☐	☒	The exact sample size (n) for each experimental group/condition, given as a discrete number and unit of measurement
☐	☒	A statement on whether measurements were taken from distinct samples or whether the same sample was measured repeatedly
☐	☒	The statistical test(s) used AND whether they are one- or two-sided<br>*Only common tests should be described solely by name; describe more complex techniques in the Methods section.*
☒	☐	A description of all covariates tested
☐	☒	A description of any assumptions or corrections, such as tests of normality and adjustment for multiple comparisons
☐	☒	A full description of the statistical parameters including central tendency (e.g. means) or other basic estimates (e.g. regression coefficient) AND variation (e.g. standard deviation) or associated estimates of uncertainty (e.g. confidence intervals)
☐	☒	For null hypothesis testing, the test statistic (e.g. F, t, r) with confidence intervals, effect sizes, degrees of freedom and P value noted<br>*Give P values as exact values whenever suitable.*
☒	☐	For Bayesian analysis, information on the choice of priors and Markov chain Monte Carlo settings
☒	☐	For hierarchical and complex designs, identification of the appropriate level for tests and full reporting of outcomes
☒	☐	Estimates of effect sizes (e.g. Cohen's d, Pearson's r), indicating how they were calculated

Our web collection on statistics for biologists contains articles on many of the points above.

Software and code

Policy information about availability of computer code

Data collection	Imaging data were captured through the Zeiss BioLite software. Flow cytometry data were collected through FlowJo, version 10.
Data analysis	No custom code was used in data analysis.<br>Imaging data were analyzed using Fiji software (ImageJ, v1.53). Flow cytometry data were analyzed using FlowJo, version 10.<br>Statistical tests for imaging and flow cytometry data were performed in Microsoft Excel, version 16.91.<br>scRNA-seq data were analyzed using CellRanger 3.1.0, and the Seurat package, version 4 in R, version 4.3.1.

For manuscripts utilizing custom algorithms or software that are central to the research but not yet described in published literature, software must be made available to editors and reviewers. We strongly encourage code deposition in a community repository (e.g. GitHub). See the Nature Portfolio guidelines for submitting code & software for further information.

Data

Policy information about availability of data

All manuscripts must include a data availability statement. This statement should provide the following information, where applicable:

- Accession codes, unique identifiers, or web links for publicly available datasets
- A description of any restrictions on data availability
- For clinical datasets or third party data, please ensure that the statement adheres to our policy

Processed and raw scRNA-seq data in this study have been deposited in the GEO database under accession code: GSE244244 https://www.ncbi.nlm.nih.gov/geo/

query/acc.cgi?acc=GSE244244.

Research involving human participants, their data, or biological material

Policy information about studies with human participants or human data. See also policy information about sex, gender (identity/presentation), and sexual orientation and race, ethnicity and racism.

Reporting on sex and gender	N/A
Reporting on race, ethnicity, or other socially relevant groupings	N/A
Population characteristics	N/A
Recruitment	N/A
Ethics oversight	N/A

Note that full information on the approval of the study protocol must also be provided in the manuscript.

Field-specific reporting

Please select the one below that is the best fit for your research. If you are not sure, read the appropriate sections before making your selection.

[x] Life sciences [ ] Behavioural & social sciences [ ] Ecological, evolutionary & environmental sciences

For a reference copy of the document with all sections, see nature.com/documents/nr-reporting-summary-flat.pdf

Life sciences study design

All studies must disclose on these points even when the disclosure is negative.

Sample size	Sample sizes were based on standards in the field. No data were included with less than three independent biological replicates per genotype, with exception to scRNA-seq. For scRNA-seq, sequencing depth was guided based on standards in the field.
Data exclusions	No data excluded from the analyses.
Replication	All imaging and flow cytometry experiments using dual-enSERT-1 mice were performed in at least four biological replicates split across two separate litters of mouse embryos.<br><br>scRNA-seq data were derived from single hindlimbs from a dual-enSERT-1 and a dual-enSERT-2 mouse embryo.<br><br>For all experiments quantifying the fluorescence of dual-enSERT-2 mouse embryos at least three independent replicates were used. For the two constructs where only single embryos were obtained, no quantification was performed. Generating more data for increasing biological replicates was cost-prohibitive.
Randomization	There was no randomization of samples in this study.
Blinding	Experimenters were blinded to genotype (transgenic versus non-transgenic) during all live imaging and flow cytometry. For quantification of imaging data, researchers were blinded to genotype of single- vs multi-copy integrants. For scRNA-seq data, blinding is not required per ENCODE guidelines.

Reporting for specific materials, systems and methods

We require information from authors about some types of materials, experimental systems and methods used in many studies. Here, indicate whether each material, system or method listed is relevant to your study. If you are not sure if a list item applies to your research, read the appropriate section before selecting a response.

Materials & experimental systems

n/a	Involved in the study
x	Antibodies
x	Eukaryotic cell lines
x	Palaeontology and archaeology
	x Animals and other organisms
x	Clinical data
x	Dual use research of concern
x	Plants

Methods

n/a	Involved in the study
x	ChIP-seq
	x Flow cytometry
x	MRI-based neuroimaging

Animals and other research organisms

Policy information about studies involving animals; ARRIVE guidelines recommended for reporting animal research, and Sex and Gender in Research

Laboratory animals	Animals used: Mus musculus; Strain: FVB/NJ and CD-1 (CD-1 mice were used as surrogate mothers). Transgenic and wild type FVB mouse embryos of embryonic age 11.5 (E11.5) were used in this study for data collection. Generally, females >6 weeks of age were used for breeding. All experiments were performed in accordance with IACUC guidelines. Mice were maintained in standard housing conditions (temperature between 65-75°C and humidity between 40-60%) on a reversed 12-hr dark–light cycle with food and water provided ad libitum. All data were generated using FVB mice.
Wild animals	No wild animals were used in this study.
Reporting on sex	Sex was not factored into study design as sex is not apparent at the stage of E11.5.
Field-collected samples	N/A
Ethics oversight	All experiments involving mice were approved by the Institutional Animal Care and Use Committee at the University of California, Irvine (approved protocol #: AUP-23-005).

Note that full information on the approval of the study protocol must also be provided in the manuscript.

Plants

Seed stocks	N/A
Novel plant genotypes	N/A
Authentication	N/A

Flow Cytometry

Plots

Confirm that:

- [x] The axis labels state the marker and fluorochrome used (e.g. CD4-FITC).
- [x] The axis scales are clearly visible. Include numbers along axes only for bottom left plot of group (a 'group' is an analysis of identical markers).
- [x] All plots are contour plots with outliers or pseudocolor plots.
- [x] A numerical value for number of cells or percentage (with statistics) is provided.

Methodology

Sample preparation	Dissected pieces of limb from each embryo were pooled separately and then incubated with collagenase II (Gibco, #17101015, 0.2 µL at 100 u/µl) for 10 min at 700 rpm and 37°C with trituration every 5 min with a P200 pipette. Then, 450 µL of 10% FBS (Thermo Fisher, #A3840201) was added and dissociated cells were spun down. Cells were resuspended in 200 µL of 0.04% BSA (Millipore Sigma, #A1595) and filtered using 40 µM P1000 Flowmi cell filters (SP Bel-Art, #136800040).

Instrument	FACSAria Fusion Sorter (BD Biosciences)
Software	FlowJo (BD Biosciences)
Cell population abundance	mCherry and eGFP gates were established by using a fluorescently-negative forebrain tissue. Any cells with signal beyond this minimum were considered double-positive, and if only exceeding one gate value, were considered single positives.
Gating strategy	Fluorescently negative forebrain tissue was utilized to establish an initial gate for mCherry and eGFP signal.

☒ Tick this box to confirm that a figure exemplifying the gating strategy is provided in the Supplementary Information.
